# Editorial of Special Issue “Molecular Mechanisms of Allergy and Asthma”

**DOI:** 10.3390/ijms222111580

**Published:** 2021-10-27

**Authors:** Daniel P. Potaczek

**Affiliations:** Translational Inflammation Research Division & Core Facility for Single Cell Multiomics, Philipps-University Marburg, 35043 Marburg, Germany; danppot@gmail.com

This Special Issue aggregates several high-quality original articles written by renowned researchers. Those articles substantially differ in their scope, which is a natural consequence of not only the broadness of the topic of the special issue, but also the multidimensional character of molecular allergology as a field.

Two studies investigated the very beginnings of allergy, starting from the perinatal period. Perveen and colleagues [1] reported protective effects of high neonatal T cell protein kinase C zeta expression against the development of allergic diseases later in life to be related to maturation of new-born T cells more towards type 1 helper T cells, with a decrease in type 9 helper T cells. Acevedo et al. [2] in turn comparatively analyzed peripheral blood mononuclear cell DNA methylation in mothers during pregnancy and their children between the birth and 5 years of age. They observed an association between maternal or offspring DNA methylation patterns and immunoglobulin E-mediated sensitization early in life. Besides, several new candidate genes for atopy were identified.

Some other papers focused on specific mechanisms of allergic airway inflammation characteristic for asthma. Bazan-Socha et al. [3] analyzed bronchial wall geometry and reticular basement membrane thickness in the context of a bronchial epithelium transcriptome profile in asthmatics. Their results suggested that being associated with a thicker reticular basement membrane, the pro-fibrotic profile in the airway epithelial cell transcriptome might contribute to asthma airway remodeling. In a methodologically robust study, Bruno and colleagues [4] demonstrated the importance of dynamin related protein 1-mediated mitochondrial fission for regulation of the pro-inflammatory response by respiratory epithelium and airway epithelial cell survival after exposure to house dust mites. Richards and co-workers [5] showed that short-chain fatty acids, fermentation metabolites from the gut microbiome, contributed to the recovery of barrier properties of airway epithelial cells, which might be mediated by increasing the expression of zonula occludens-1, a tight junction protein contributing to the integrity of the epithelium. In turn, van Heerden et al. [6] showed that long, asthma-associated form of thymic stromal lymphopoietin downregulated the production of immunoglobulin A, which might negatively affect the surveillance of mucosal surfaces in asthma. Amniai et al. [7] demonstrated that natural killer cells from allergic donors were defective in their response to CC chemokine ligand 18, a CC chemokine constitutively highly expressed in human lungs and plasma and upregulated during inflammatory and cancer processes in many organs, which might participate in the defect in the natural killer cell activation observed in asthma. In a mouse study, MacBeth et al. [8] showed that alterations in transcription factor binding and a novel variable dinucleotide repeat of variable length impacted the regulation of interleukin-6, contributing to naturally occurring regulatory T cell plasticity important for interleukin-6-related disease with impaired function of naturally occurring regulatory T cells. Finally, Alhamdan and colleagues [9] investigated genome-wide expression patterns in purified CD4+ T cells obtained from asthmatic adults with or without obesity and healthy controls. They found interferon-related signaling pathways to be specifically overrepresented in obese asthmatics, while the gap junction and G protein-coupled receptor ligand binding pathways were enriched in both asthma groups. The topological interconnection between interferon signaling and viral infection pathways in subjects with low type-2 obesity-associated asthma might define a complex mechanism underlying this phenotype.

Other allergic disorders have also been targeted. Bélanger and colleagues [10] sought to find a differential microRNA pattern in eosinophils from patients presenting not only with asthma but also other diseases included in the atopic march process, such as allergic rhinitis or atopic dermatitis, and unaffected individuals. They found eighteen microRNAs to be differentially expressed. Moreover, counts from these microRNAs made it possible to cluster study individuals into groups that could be explained by the clinical characteristics of the patients. Franke et al. [11] demonstrated interleukin-33 to generally foster the cytokine producing apparatus of skin mast cells through operation at different levels and in a potent cooperation with the high affinity immunoglobulin E receptor and MAS-related G protein-coupled receptor X2. This suggested the role of interleukin-33 as an efficient amplifier of skin inflammation in inflammatory dermatoses such as atopic dermatitis, urticaria and psoriasis. The data obtained by Nieto et al. [12] indicated a novel mechanism involved in controlling ocular allergic diseases, with α-melanocyte stimulating hormone diminishing the concentration of interleukin-6 and -4, thus restoring the frequency of regulatory T cells and down-regulating CD4+ T cell activation. In addition, they demonstrated the involvement of CD4+TLR4+ cells as an effector cell subset in allergic conjunctivitis.

Several further studies addressed food allergies or allergens. In a mouse model, Abbring et al. [13] demonstrated raw milk-induced protection against food allergy symptoms to be accompanied by shifts in the gut microbiome. Siekierzynska and colleagues [14] assessed whether factors such as the cultivation method, maturity stage, genotype, or type of tissue might place an impact on the allergenic potential of apples. Their results made it possible to distinguish between potentially low, medium, and highly allergenic apple varieties. Mattison et al. [15] performed a microbiological, physicochemical, and immunological analysis of a commercial cashew nut-based yogurt, which indicated that it was not suitable for individuals with a cashew nut allergy. Iweala and coworkers [16] investigated, in turn, a unique type of allergy. They demonstrated that in alpha-gal syndrome, peripheral blood T and B lymphocyte transcriptional states could differentiate between sensitized and sensitized individuals.

Finally, this Special Issue comprises also a small set and state-of-the art review articles. While Nagata and Nishiyama [17] outlined the anti- and proinflammatory roles of interleukin-10 in mast cell-mediated immune responses, Sroka-Tomaszewska and Trzeciak [18] summarized and discussed the molecular mechanisms underlying atopic dermatitis. The other three reviews referred to asthma. Whereas the article by di Palmo and colleagues [19] addressed the predictive role of biomarkers and genetics in childhood asthma exacerbations, the work by Chiu and Huang [20] summarized the current state of knowledge on the role of biologics and probiotics in asthma. Finally, Alashkar Alhamwe et al. [21] outlined and discussed the knowledge on the role of extracellular vesicles in asthma.

I strongly believe that the articles belonging to this Special Issue are of interest to many scientists and medical practitioners dealing with asthma and/or other allergies.
